# The interplay between doxorubicin chemotherapy, antioxidant system, and cardiotoxicity: Unrevealing of the protective potential of tannic acid

**DOI:** 10.1002/bab.2648

**Published:** 2024-08-05

**Authors:** Guldemet Kansu, Neslihan Ozturk, Medine Sibel Karagac, Esra Nur Yesilkent, Hamid Ceylan

**Affiliations:** ^1^ Department of Molecular Biology and Genetics, Faculty of Science Atatürk University Erzurum Türkiye

**Keywords:** antioxidant system, cardiotoxicity, doxorubicin, heart, tannic acid

## Abstract

Cardiotoxicity is the leading side effect of anthracycline‐based chemotherapy. Therefore, it has gained importance to reveal chemotherapy‐supporting strategies and reliable agents with their mechanisms of action. Tannic acid (TA), a naturally occurring plant polyphenol, has diverse physiological effects, including anti‐inflammatory, anticarcinogenic, antioxidant, and radical scavenging properties. Therefore, this study was designed to investigate whether TA exerts a protective effect on mechanisms contributing to anthracycline‐induced cardiotoxicity in rat heart tissues exposed to doxorubicin (DOX). Rats were randomly divided into control and experimental groups and treated with (18 mg/kg) DOX, TA (50 mg/kg), and DOX + TA during the 2 weeks. Cardiac gene markers and mitochondrial DNA (mtDNA) content were evaluated in the heart tissues of all animals. In addition to major metabolites, mRNA expression changes and biological activity responses of components of antioxidant metabolism were examined in the heart tissues of all animals. The expression of cardiac gene markers increased by DOX exposure was significantly reduced by TA treatment, whereas mtDNA content, which was decreased by DOX exposure, was significantly increased. TA also improved antioxidant metabolism members' gene expression and enzymatic activity, including glutathione peroxidase, glutathione s‐transferase, superoxide dismutase, catalase, and thioredoxin reductase. This study provides a detailed overview of the current understanding of DOX‐induced cardiotoxicity and preventive or curative measures involving TA.

**Abbreviations**
ANP: atrial natriuretic peptide
BNP: B‐type natriuretic peptide
CAT: catalase
cDNAs: complementary DNAs
DOX: doxorubicin
GPx: glutathione peroxidase
GSH: glutathione
GST: glutathione *s*‐transferase
MDA: malondialdehyde
MF: myocardial fibrosis
mtDNA: mitochondrial DNA
ROS: reactive oxygen species
SOD: superoxide dismutase
TA: tannic acid
Trx: thioredoxin
TRXR: thioredoxin reductase

## 1 INTRODUCTION

Doxorubicin (DOX) is a potent chemotherapeutic agent used in the treatment of various malignancies. Despite its effectiveness, the clinical use of DOX is significantly limited by its dose‐dependent cardiotoxic side effects, which can lead to irreversible heart damage.^1^, ^2^, ^3^ Clinically, DOX‐induced cardiotoxicity is characterized by a decrease in left ventricular ejection fraction, arrhythmias, and congestive heart failure.^4^ These manifestations can occur acutely, within days of administration, or chronically, developing months to years after treatment. The relationship between DOX and cardiotoxicity is complex and multifaceted, involving various cellular mechanisms and pathways.^5^, ^6^

The heart's vulnerability to DOX is primarily attributed to the generation of reactive oxygen species (ROS) and oxidative stress caused by the drug, which overwhelms the body's natural antioxidant defenses and damages cellular components, including lipids, proteins, and DNA.^7^, ^8^, ^9^ The antioxidant system, which includes enzymes like glutathione peroxidase (GPx), superoxide dismutase (SOD), and catalase (CAT), plays a critical role in neutralizing ROS and protecting the body from oxidative damage.^10^, ^11^ However, the heart is particularly susceptible to oxidative stress due to its high metabolic requirement and relatively low antioxidant capacity.^12^ The former leads to increased ROS production and mitochondrial DNA (mtDNA) damage due to disruption of the mitochondrial electron transport chain,^13^ whereas the latter contributes to the deterioration of heart function by leading to the death of cardiomyocytes.^14^

Given the growing population of cancer survivors and the interdisciplinary interest in DOX cardiotoxicity, there is an urgent need to design successful preventive or curative measures. Current strategies include limiting the cumulative dose of DOX^15^ and using cardioprotective agents.^16^ Although many reviews suggest that limiting the cumulative DOX dose can exterminate acute and early‐onset chronic myocardial degeneration, this creates a dilemma as it moves oncological treatment away from optimal efficiency.^17^ Therefore, in recent years, the detection and use of prophylactic drugs against DOX cardiotoxicity have been accepted as a promising research area in cardio‐oncology.^18^ Phytocompounds such as flavonoids and polyphenols offer a potential strategy to improve the quality of life of cancer patients undergoing chemotherapy due to their properties, such as antioxidant activity, anti‐inflammatory effects, modulation of apoptosis, and protection of mitochondrial integrity.^19^, ^20^ Studies have shown that tannic acid (TA), a type of plant‐based polyphenol, can prevent pathologies caused by oxidative burden, especially by eliminating free radicals formed in heart tissue.^21^, ^22^ Researchers have examined its protective ability in diverse contexts, including cardiotoxicity. Studies suggest that TA may ameliorate DOX‐associated cardiotoxicity by enhancing cellular antioxidant capacity, depressing inflammation, and apoptosis.^23^, ^24^

Understanding the complex interplay between DOX metabolism, mechanisms of action, and cardiotoxicity drivers is crucial. Based on extracted data from previous important findings, it can be assumed that TA can be considered a promising candidate for therapeutic action in DOX cardiotoxicity. Therefore, in this study, we aimed to examine the mechanisms involved in DOX‐related cardiotoxicity. Further, the effect of DOX and TA‐DOX was compared in rat heart tissue.

## 2 MATERIALS AND METHODS

### 2.1 Animals and study design

**FIGURE 1:**
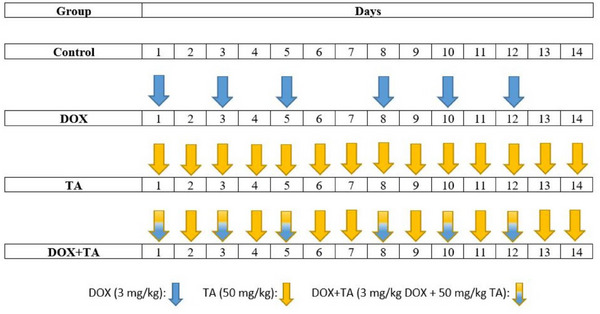
In vivo experimental study protocol. CON group was injected with saline (placebo) daily. Doxorubicin (DOX) injection was performed on Days 1, 3, 5, 8, 10, and 12. The tannic acid (TA) group was injected with TA for 14 consecutive days. The same administration as the DOX and TA groups was performed on the DOX + TA group.

The experimental protocol is presented in Figure 1. Animals (Sprague‐Dawley rats, *Rattus norvegicus*, male, 180 ± 10 g, *n* = 20) were randomly divided into four groups. To mimic the human chemotherapy protocol, a cumulative DOX dose of 18 mg/kg was administered to DOX group rats through six equal (3 mg/kg) intraperitoneal injections (i.p.) on different days.^19^, ^25^ On non‐injection days, normal saline was injected (i.p.). Negative control (CON) rats were injected with saline (i.p.) for 14 consecutive days. TA group rats were injected with 50 mg/kg TA (i.p.) for 14 days.^10^ TA was administered to the rats in the combined group, where DOX and TA were administered together, 1 h before DOX injection.^26^ After 14 days, ketamine/xylazine cocktail was used to anesthetize rats; hearts were excised quickly, washed in ice‐cold PBS, and stored at −70°C for further experiments. Rats were obtained from the Atatürk University Medical Experimental Application and Research Center and were kept under standard conditions (ad libitum, 22 ± 1°C air conditioning, 60% humidity, and 12–12 h lighting conditions) during the study. All animal experiments were performed under the National Research Council's Guide for the Care and Use of Laboratory Animals and were approved by the Atatürk University Local Ethics Council for Animal Experiments (Protocol No.: 2022‐10/1945).

### 2.2 Assessment of oxidative stress indicators

To examine the oxidative stress status after DOX exposure and TA treatment in the heart tissues of untreated and other experimental rat groups, malondialdehyde (MDA; secondary products of lipid peroxidation) levels and glutathione (GSH) contents, which are biomarkers of oxidative stress, were measured. MDA levels in rat heart tissue were measured at wavelengths of 532 nm according to the thiobarbituric acid method described by Suleyman et al.^27^ and presented as nanomoles of MDA per milligram protein. The reduced GSH quantity in tissue samples was measured at wavelengths of 450 nm, as previously described.^28^

### 2.3 Measurements of the antioxidant enzyme activities

Total protein was determined by the Bradford method,^29^ which is based on measuring the absorbance of the complex formed by Coomassie Brilliant Blue G 250 dye with protein at 595 nm wavelength using BSA (1 mg/mL) as a standard. To measure GPx enzymatic activity, 100 mg of heart tissues were homogenized (Heidolph Silent Crusher M) in a buffer containing 1 mM EDTA, 1 mM DDT, 1 mM PMSF, and 50 mM Tris hydrochloric acid. The absorbance was measured at 340 nm. To measure glutathione *s*‐transferase (GST) enzymatic activity, the optical density of 10 µL of supernatant of heart tissue homogenate, 20 mM GSH, and 100 mM phosphate buffer contained mixture was measured at 340 nm after adding 6 mM NADP^+^.^30^ Enzymatic activity of SOD was examined by the nitroblue tetrazolium chloride method.^31^ The absorbance was measured at 532 nm. Finally, CAT enzyme activity was measured by following the Aebi method modified by Kocpinar et al.^28^ Thioredoxin reductase (TRXR) enzyme measurement was based on the fact that TRXR catalyzes the nicotinamide adenine dinucleotide–dependent reduction of disulfide bonds in 5,5′‐dithiobis‐(2‐nitrobenzoic acid) (DTNB). TRXR enzyme activity was measured by modifying the method by Arner and Holmgren.^32^ A microplate reader (Multiskan GO, Thermo Scientific) was used for all absorbance measurements.

### 2.4 RNA isolation and cDNA library construction

RNA isolation from heart tissues was performed by the Trizol method.^33^ Approximately 40 mg of tissue was fragmented in a homogenizer with 500 µL Trizol, purified from protein and DNA, and thawed by adding sterile dH_2_O. The purity levels (A260/A280) and concentrations of RNAs were determined quantitatively with the NanoDrop device (Multiskan GO, Thermo Scientific). The concentrations of the RNAs were equalized before the synthesis process to ensure that all synthesized complementary DNAs (cDNAs) were at equal concentrations. cDNA synthesis was performed using a commercial kit (Bio‐Rad, iScript cDNA Synthesis Kit). The synthesis was performed following the protocol recommended by the manufacturer. After the synthesis reaction was terminated, the products obtained were stored at −20°C to be used in quantitative PCR (qPCR) analysis.

### 2.5 Primer design and quantitative real‐time PCR (qPCR)

For the design of unique primer sequences, sequence information of target genes was obtained from the online database https://www.ncbi.nlm.nih.gov/ (accessed on July 16, 2024). Primer sequences with the desired properties were determined using the online software http://bioinfo.ut.ee/primer3‐0.4.0/ (accessed on July 16, 2024). The binding specificities of the determined primer sequences were confirmed using the https://blast.ncbi.nlm.nih.gov/Blast.cgi (accessed on July 16, 2024) module. All primers are listed in Table 1. qPCR analysis was performed following the SYBR Green protocol (Bio‐Rad, SsoAdvanced Universal SYBR Green Supermix). The ΔCt method was used to evaluate the obtained Ct values.^34^ *Gapdh* (glyceraldehyde‐3‐phosphate dehydrogenase) was used as the reference (housekeeping) gene for normalization.

**TABLE 1:** Sequences of primer sets used in quantitative PCR (qPCR).

Gene symbol	Accession number	Sequence	*T*_m_ (°C)
*Gpx_F*	NM_030826.4	5′‐TCGGACATCAGGAGAATGG‐3′	59.57
*Gpx_R*		5′‐AGGTAAAGAGCGGGTGAGC‐3′	59.44
*Gst_F*	NM_001010921.1	5′‐TTCTGACCCCTTTCCCTCTG‐3′	59.67
*Gst_R*		5′‐TGGCTGGCTTTCTCTGACTG‐3′	59.97
*Cat_F*	NM_012520.2	5′‐ACATGGTCTGGGACTTCTGG‐3′	59.96
*Cat_R*		5′‐CCATTCGCATTAACCAGCTT‐3′	60.10
*Sod_F*	NM_017050.1	5′‐GGTCCACGAGAAACAAGATGA‐3′	60.10
*Sod_R*		5′‐CAATCACACCACAAGCCAAG‐3′	60.15
*Txnrd2_F*	NM_022584.3	5′‐AAGCCGTGCAAAACCATGTG‐3′	59.97
*Txnrd2_R*		5′‐ACCGTGAACTGTGTGCTCGT‐3′	60.04
*D‐loop_F*	X52757.1	5′‐AGGCATCTGGTTCTTACTTCAG‐3′	59.91
*D‐loop_R*		5′‐TGACGGCTATGTTGAGGAAG‐3′	59.69
*18SrRNA_F*	M11188.1	5′‐GACTCAACACGGGAAACCTC‐3′	59.55
*18SrRNA_R*		5′‐TAACCAGACAAATCGCTCCA‐3′	59.27
*β‐Mhc_F*	NM_017240.2	5′‐TTGATGTGCTGGGCTTCAC‐3′	60.42
*β‐Mhc_R*		5′‐CTCCTCCCTCTGCTTCTGTT–3′	59.98
*Anp_F*	M27498.1	5′‐AGAGAGTGAGCCGAGACAGC‐3′	59.89
*Anp_R*		5′‐AGCCCTTGGTGATGGAGAA‐3′	60.61
*Bnp_F*	M25297.1	5′‐ACAAGAGAGAGCAGGACACC‐3′	59.12
*Bnp_R*		5′‐AAAGCAGGAGCAGAATCATC‐3′	59.20
*Gapdh_F*	NM_017008.3	5′‐AAACCCATCACCATCTTCCA‐3′	60.17
*Gapdh_R*		5′‐ATACTCAGCACCAGCATCACC‐3′	60.16

Abbreviations: F, forward; R, reverse; *T*_m_, melting temperature.

### 2.6 Determination of mtDNA copy number

mtDNA count is used as an indirect marker of mitochondrial function. For this purpose, first, genomic DNA was isolated with the help of a commercial kit (QIAamp DNA Mini Kit; Qiagen). In the qPCR process, 18S rRNA primers were used as the nuclear target, and primers specifically designed for the mitochondrial D‐loop region were used as the mtDNA target (Table 1). Analysis of the mtDNA/nDNA ratio was analyzed by calculating the number of mtDNA molecules per nDNA molecule.^35^

### 2.7 The qPCR detection of the cardiac gene markers

To test whether DOX causes damage to the heart tissue and whether TA reduces the damage, mRNA expression levels of cardiac gene markers associated with cardiomyopathies or cardiac toxicity (atrial natriuretic peptide prohormone, *Anp*; also known as *Nppa*, natriuretic peptide precursor B; *Bnp*, also known as *Nppb*; and myosin, heavy chain 7, cardiac muscle, Beta; *β‐Mhc*)^36^, ^37^ were measured by qPCR. The ΔCt method was used to evaluate the obtained Ct values.^34^ *Gapdh* (glyceraldehyde‐3‐phosphate dehydrogenase) was used as the reference (housekeeping) gene for normalization.

### 2.8 Statistical analysis

Statistical comparison of data obtained from measurements made in triplicate (for each animal and sample) was evaluated with one‐way ANOVA and Tukey's post hoc test using Prism (GraphPad Software) software. The statistically significant differences are presented as follows: ^ns^ *p* > 0.05 (not significant compared to the control group); *p < 0.05 (significant); **p < 0.01 (very significant); *** or **** *p *< 0.001 or 0.001 (extremely significant).

## 3 RESULTS

### 3.1 Effects of DOX and TA on the expression levels of cardiac gene markers and mtDNA content

**FIGURE 2:**
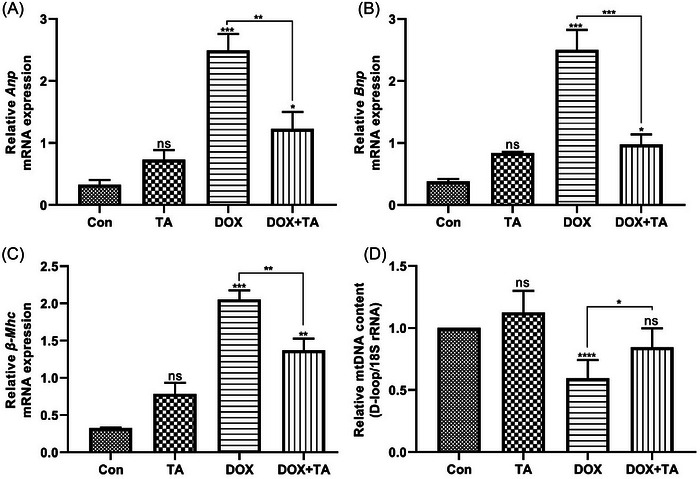
mRNA expression of cardiac gene markers in the heart tissues of the control versus treated groups. (A) The relative mRNA expression levels of *Anp*. (B) The relative mRNA expression levels of *Bnp*. (C) The relative mRNA expression levels of *β‐Mhc*. (D) Mitochondrial DNA (mtDNA) copy number after doxorubicin (DOX) or tannic acid (TA) treatment, quantified by quantitative PCR (qPCR) relative to the nuclear genome. “ns” represents *p* > 0.05, “*” represents *p* < 0.05, “**” represents *p* < 0.01, “***” represents *p* < 0.001, and “****” represents *p* < 0.0001 versus control group. The data are shown as mean ± SEM (*n* = 5).

First, to understand whether DOX harms normal cardiac functioning as well as to test the protection provided by TA against DOX, the change in mRNA expression levels of cardiac damage genes was detected by qPCR. The results showed that mRNA levels of damage markers were increased significantly in the heart tissue of DOX‐injected rats compared with the control group. However, it was observed that the expression of these genes decreased significantly with TA treatment and was repressed markedly compared to DOX alone (Figure 2A–C). These findings prove that TA can improve cardiac functions and delay heart defect progression after DOX. mtDNA copy number decreased significantly in the DOX group compared to untreated and TA‐treated groups. However, the mtDNA content tended to increase in the heart tissues of rats administered TA simultaneously with DOX (Figure 2D). The combination of high damage marker gene expression and depletion of mtDNA content is extremely suggestive that DOX causes cardiac damage. It appears that in the presence of TA, mRNA levels of damage marker genes decreased and mtDNA content increased; therefore, DOX‐induced damage may be reduced through TA.

### 3.2 Effects of DOX and TA administration on heart MDA and GSH content

**FIGURE 3:**
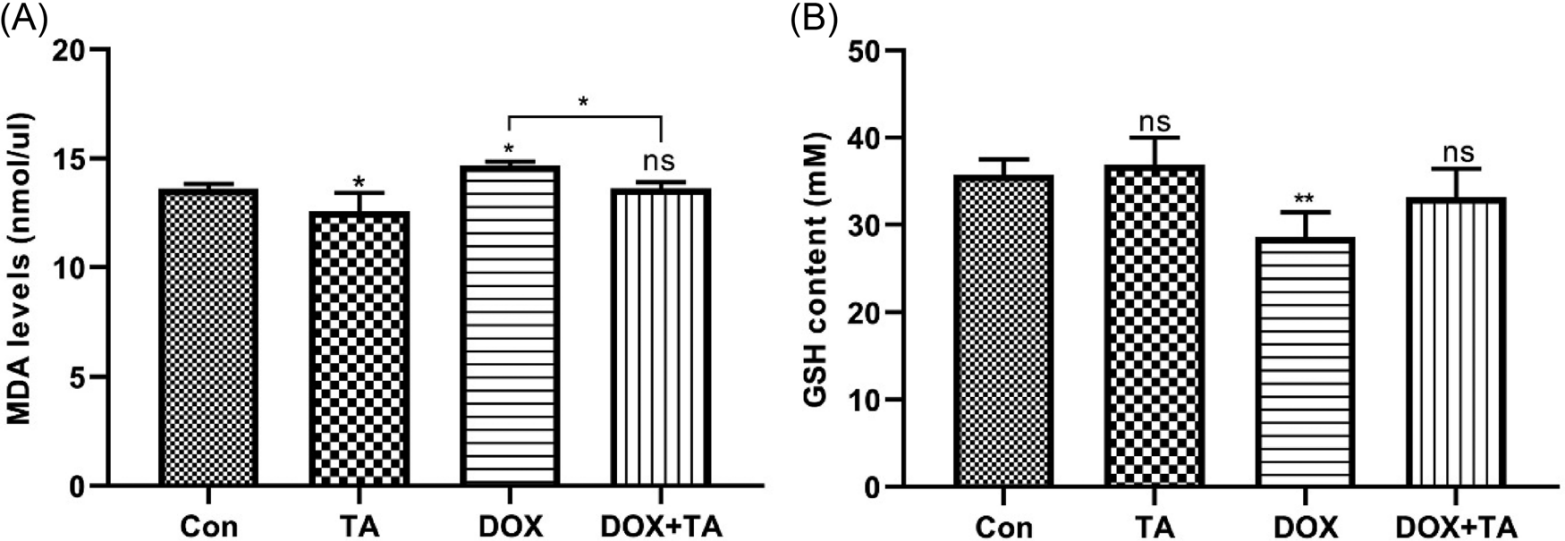
Malondialdehyde (MDA) levels and reduced glutathione (GSH) content in the rat heart tissues. MDA levels in liver tissues (A) and comparison of GSH content (B). “ns” represents *p* > 0.05, “*” represents *p* < 0.05, and “**” represents *p* < 0.01 versus control group. The data are shown as mean ± SEM (*n* = 5).

To investigate the impact of DOX and TA on rat heart redox balance, assessments were made on lipid peroxidation and total GSH content. Initially, the MDA concentration was measured in the hearts of rats subjected to DOX treatment, both solely and in conjunction with TA. As depicted in Figure 3A, a notable elevation in MDA levels was observed in the hearts of rats within the DOX‐only group, relative to the control group. Conversely, TA administration by itself did not lead to a marked rise in MDA levels. Furthermore, TA effectively mitigated the rise in lipid peroxidation prompted by DOX. Upon examining GSH levels, it was noted that DOX treatment alone significantly diminished the total GSH content, whereas TA on its own had no impact on GSH levels (Figure 3B). Additionally, the concurrent administration of TA with DOX reversed the reduction in GSH levels caused by DOX.

### 3.3 Antioxidant system status of heart tissue after DOX and TA administration

**FIGURE 4:**
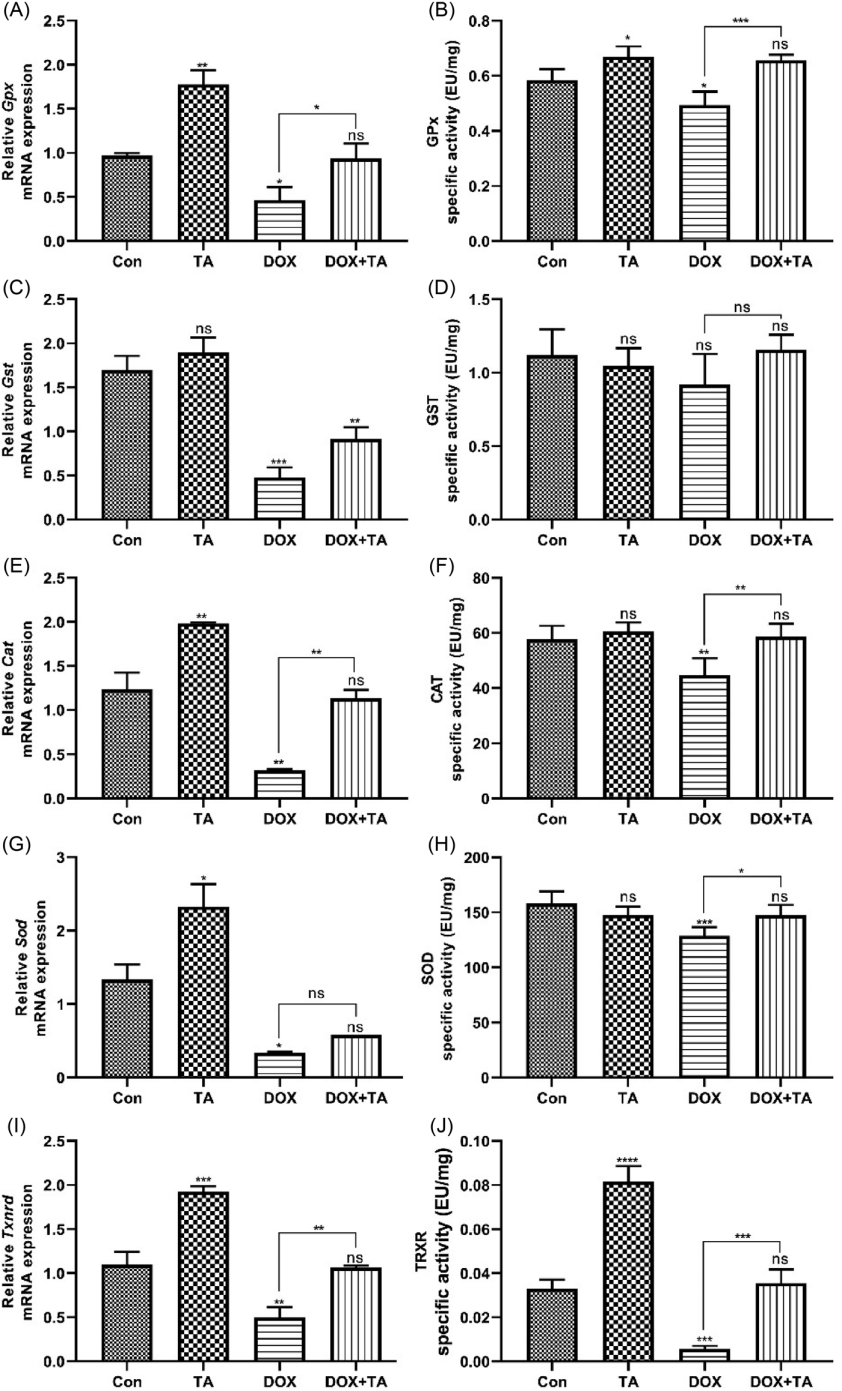
Effects of doxorubicin (DOX) and tannic acid (TA) on the mRNA expression and specific activities of antioxidant metabolism members in the rat heart tissues. The relative mRNA expressions of Gpx, glutathione peroxidase (A); *Gst*, glutathione *s*‐transferase (C); *Cat*, catalase (E); *Sod*, superoxide dismutase (G); and *Txnrd2*, thioredoxin reductase 2 (I). The enzymatic activities of GPx (B), GST (D), CAT (F), SOD (H), and TRXR (J) after saline, TA, DOX, and DOX + TA treatment. “ns” represents *p* > 0.05, “*” represents *p* < 0.05, “**” represents *p* < 0.01, “***” represents *p* < 0.001, and “****” represents *p* < 0.0001 versus control group. The data are shown as mean ± SEM (*n* = 5).

The impact of DOX and TA on antioxidant system components was examined at both gene and protein levels. As depicted in Figure 4A,B, the notable reduction in *Gpx* mRNA expression induced by DOX was counteracted with TA treatment. A similar trend was observed in GPx enzyme activity. The DOX‐induced loss of enzyme activity was significantly recovered by TA, reaching the control group's level. *Gst* mRNA expression was significantly diminished by DOX exposure, but TA administration did not affect it. Furthermore, the combination of DOX + TA significantly prevented the severe reduction in gene expression compared to DOX administration alone (Figure 4C). It was also noted that GST enzymatic activity significantly decreased after DOX administration (Figure 4D). Comparable outcomes were found for CAT and SOD. *Cat* and *Sod* mRNA expression, which was suppressed by DOX exposure alone, rebounded with TA supplementation (Figure 4E,G). *Cat* and *Sod* mRNA expression were significantly inhibited with DOX. However, especially the *Cat* mRNA level, significantly rebounded with TA treatment compared to DOX exposure alone. It was noted that CAT and SOD enzyme activities were significantly reduced after DOX. However, TA supplementation steadily increased enzyme activities, especially CAT (Figure 4F,H). Lastly, the quantification of mitochondrial TRXR mRNA and enzyme activity were evaluated. The mRNA expression of *Txnrd2* was significantly suppressed after DOX exposure. However, the simultaneous administration of TA with DOX reversed the DOX‐induced reduction in gene expression (Figure 4I). Similar outcomes were found for TRXR activity. It was noted that the enzyme activity, which was significantly suppressed by DOX application, rebounded with TA treatment (Figure 4J). These findings suggest that TA may protect heart tissue from DOX‐induced oxidative stress by enhancing the heart's antioxidant capacity.

## 4 DISCUSSION

As it is known, cancer remains a global health problem affecting millions of lives today and significantly affects the quality of life of patients due to the difficulties in diagnosis and treatment.^38^ Traditional treatment strategies, such as radiotherapy, surgery, and chemotherapy, have limited effectiveness and can cause serious side effects that affect the patient's post‐treatment life.^39^ In this context, the use of supportive agents that do not limit treatment effectiveness during and after cancer treatment is accepted as a rational approach.^40^ Phytotherapy, which includes the use of plant‐based compounds, has attracted attention in recent years as a complementary approach to increase patient well‐being by minimizing the negativities that may occur during and after treatment.^41^ As a matter of fact, due to modern phytotherapy, which is seen as a part of evidence‐based medicine, almost half of the phytopharmaceutical users are cancer patients. The current study was conducted to explore the cardiotoxic activity of DOX and the protective potential of TA on rat heart tissues. To achieve this goal, the effect of TA on the mtDNA content in the heart tissue of rats treated with DOX, as well as the mRNA expressions and biological activities of the major players involved in the antioxidant mechanism, were profiled.

Cardiotoxicity is one of the important side effects that may occur with radiation therapy as well as chemotherapeutic drugs administered to patients in cancer treatment.^42^ The imbalance occurring in mitochondrial dynamics causes cardiotoxicity by triggering mechanisms, such as oxidative stress,^43^ inflammation,^44^ fibrosis, and autophagy dysregulation,^45^ which affect processes, such as energy metabolism management, apoptosis, and ROS production. Therefore, mitochondria, which are responsible for the cell's energy production, are the primary target in cardiotoxicity caused by anthracycline group therapeutics, especially DOX.^46^ In cancer, where normal mitochondrial functioning is compromised, chemotherapy‐induced oxidative stress may become more severe and contribute to cardiotoxicity. mtDNA content results in insufficient synthesis of mitochondrial respiratory chain complexes, which provide the energy the cell needs to combat chemotherapy‐induced oxidative stress.^47^ Therefore, it is very important to identify strategies to prevent mtDNA depletion to minimize the risk of chemotherapy‐induced cardiotoxicity. A wide variety of studies have demonstrated that increasing mtDNA copy number has been a proposed approach to alleviate DOX‐induced cardiotoxicity. Lebrecht et al.^48^ reported that protecting cardiac mitochondria may prevent DOX‐induced cardiomyopathy by preventing cardiomyocyte mtDNA copy number reduction and mtDNA deletions. Another study has indicated that long‐term DOX exposure causes cardiotoxicity by decreasing the activities of respiratory chain enzymes encoded by the mitochondrial genome due to decreasing mtDNA levels and increasing common mtDNA deletions.^49^ In this context, we first examined the effect of DOX and TA on the mtDNA content of the rat heart tissues. Our results showed that TA altered mtDNA copy number in rat heart tissues under DOX treatment. Based on these observations, we speculated that preserving mtDNA content with TA may be highly effective to combat cardiotoxicity occurring in the presence of DOX.

The enzymatic activity of cellular antioxidant system compounds is boosted to prevent toxic outcomes caused by redox balance disruption due to excess ROS to maintain cell viability.^50^, ^51^ The thioredoxin (Trx) system, which is an important component of the antioxidant system and includes Trx and TRXR, can convert excess ROS into harmless components.^52^ Berggren et al.^53^ showed that increased Trx expression could provide enhanced protection by removing H_2_O_2_ by triggering peroxiredoxin (*Prx*) overexpression. Another study also demonstrated that upregulated Trx‐1 displays protection against adriamycin‐induced (a member of anthracyclines) cardiotoxicity by increasing the buffering capacity of antioxidant defenses and reducing oxidative stress.^54^ These findings strongly suggest that targeting the Trx system is an effective strategy to reorganize the redox imbalance caused by DOX chemotherapy. Consistent with these findings, our current results show that TA can abolish the inhibitory effect of DOX on components of the Trx system and major antioxidant actors, including GPx, GST, SOD, and CAT.

Myocardial fibrosis (MF) is a serious pathological change that is very important for myocardial remodeling and can ultimately lead to cardiovascular diseases (CVDs) such as heart failure.^55^, ^56^ Atrial natriuretic peptide (ANP) and B‐type natriuretic peptide (BNP) are two important cardiac peptides associated with MF and have effects on the myocardium such as hypertrophy, cardiomyopathy, fibrosis, and arrhythmia.^57^ It has been shown by Ma et al.^23^ that expression levels of *ANP* and *BNP* increased dramatically in isoproterenol (ISO)‐induced cardiac injury mice heart tissues. Surprisingly, myocardial ANP and BNP expressions were reversed by TA administration, thus suggesting that TA could inhibit MF expression of these peptides. Consistent with these findings, the results obtained from the current study show that DOX‐induced overexpression of these peptides can be effectively suppressed by TA. Overall, we can speculate that TA exerts notable cardioprotective effects in DOX‐induced cardiac fibrosis.

In summary, rational integrative oncology recommends the judicious use of well‐defined phytopharmaceuticals during DOX chemotherapy to improve patient well‐being. By combining evidence‐based treatments with the power of nature‐based compounds, we can contribute to optimizing treatment outcomes while minimizing adverse effects from chemotherapy.

## 5 CONCLUSION

Although the mechanisms underlying DOX‐induced cardiac injury are becoming clearer, preventing and mitigating adverse effects is a source of significant concern in the field of cardio‐oncology, and much remains to be learned. This study provides a detailed overview of the current understanding of DOX‐induced cardiotoxicity and preventive or curative measures. However, further in‐depth exploration, interdisciplinary collaboration, and human studies are needed to improve the quality of life for cancer survivors.

## AUTHOR CONTRIBUTIONS

**Hamid Ceylan**: Conceptualization and design; data curation; formal analysis; writing—original draft. **Guldemet Kansu**: **Neslihan Ozturk**: **Medine Sibel Karagac**: and **Esra Nur Yesilkent**: Data curation; formal analysis; animal care; experiments. All authors contributed to the article and approved the submitted version.

## CONFLICT OF INTEREST STATEMENT

The authors declare no conflicts of interest.

7

## CONSENT TO PARTICIPATE

This article does not contain any studies involving human participants performed by any of the authors.

## CONSENT TO PUBLISH

All authors have given their consent to publish this work.

## Data Availability

The data used and analyzed during the current study are available from the corresponding author upon reasonable request.
